# Estimating the Richness of a Population When the Maximum Number of Classes Is Fixed: A Nonparametric Solution to an Archaeological Problem

**DOI:** 10.1371/journal.pone.0034179

**Published:** 2012-05-29

**Authors:** Metin I. Eren, Anne Chao, Wen-Han Hwang, Robert K. Colwell

**Affiliations:** 1 Department of Anthropology, University of Kent, Canterbury, United Kingdom; 2 Institute of Statistics, National Tsing Hua University, Hsin-Chu, Taiwan; 3 Institute of Statistics, National Chung Hsing University, Taichung, Taiwan; 4 Department of Ecology and Evolutionary Biology, University of Connecticut, Storrs, Connecticut, United States of America; Queen Mary, University of London, United Kingdom

## Abstract

**Background:**

Estimating assemblage species or class richness from samples remains a challenging, but essential, goal. Though a variety of statistical tools for estimating species or class richness have been developed, they are all singly-bounded: assuming only a lower bound of species or classes. Nevertheless there are numerous situations, particularly in the cultural realm, where the maximum number of classes is fixed. For this reason, a new method is needed to estimate richness when both upper and lower bounds are known.

**Methodology/Principal Findings:**

Here, we introduce a new method for estimating class richness: doubly-bounded confidence intervals (both lower and upper bounds are known). We specifically illustrate our new method using the Chao1 estimator, rarefaction, and extrapolation, although any estimator of asymptotic richness can be used in our method. Using a case study of Clovis stone tools from the North American Lower Great Lakes region, we demonstrate that singly-bounded richness estimators can yield confidence intervals with upper bound estimates larger than the possible maximum number of classes, while our new method provides estimates that make empirical sense.

**Conclusions/Significance:**

Application of the new method for constructing doubly-bound richness estimates of Clovis stone tools permitted conclusions to be drawn that were not otherwise possible with singly-bounded richness estimates, namely, that Lower Great Lakes Clovis Paleoindians utilized a settlement pattern that was probably more logistical in nature than residential. However, our new method is not limited to archaeological applications. It can be applied to any set of data for which there is a fixed maximum number of classes, whether that be site occupancy models, commercial products (e.g. athletic shoes), or census information (e.g. nationality, religion, age, race).

## Introduction

The concept of richness, defined as the number of species or classes in a biological assemblage, is the simplest and the most intuitive concept for characterizing assemblage (community) diversity ([1];[2];[3];[4]). The measurement of richness, however, is not always straightforward ([5]). Researchers who sample biological assemblages must face the problem of how well a sample reflects a community’s “true” (asymptotic) richness ([6];[7]). For this reason, extrapolating from the known to the unknown is now an essential objective in ecology, paleontology, and conservation biology ([8]). For this reason, a variety of statistical tools for estimating species or class richness have been developed, including rarefaction ([4];[9];[10];[11];[12]), extrapolation from accumulation curves ([3]), parametric estimators ([13]), and nonparametric estimators (e.g. [2];[14]).

In ecological and biogeographic assessments of richness, established upper limits for the number of species that can be found in a particular region are rarely, if ever, known. This is because species can immigrate, emigrate, speciate, become extinct, hide, get lost, or simply be too rare to be observed with practical levels of sampling effort. New species are constantly being discovered (e.g. [15]), even primates ([16]). There are always more species lurking somewhere in a study region, even if just vagrants from elsewhere. As such, biological richness estimators have been universally constructed without a known upper bound as a constraint. In contrast, most richness estimators have a lower bound set, sensibly enough, by the observed number of species or classes.

For the past thirty years it has been commonplace for archaeologists to apply these singly-bounded (a lower, but no upper bound) richness estimators to samples of stone artifacts in order to estimate the “true” artifact richness of an assemblage (e.g. [17];[18];[19];[20];[21];[22];[23], and papers therein; [24];[25]). Archaeologists often treat stone tools like biological entities, in the sense that new classes ( =  species) can always be discovered (e.g. [26];[27];[28];[29]). Fieldwork and excavation in new geographic areas and/or time periods may yield unique, novel forms. Moreover, with an increased understanding of stone tool production techniques (called flintknapping) and tool uses, new “technological” and “functional” classes that previously went unnoticed can be discovered and described by reexamining previously studied artifact assemblages (e.g. [30];[31];[32]). In this sense, there is no logical incongruity in the application of singly-bounded richness estimators to archaeological stone tools.

**Figure 1:**
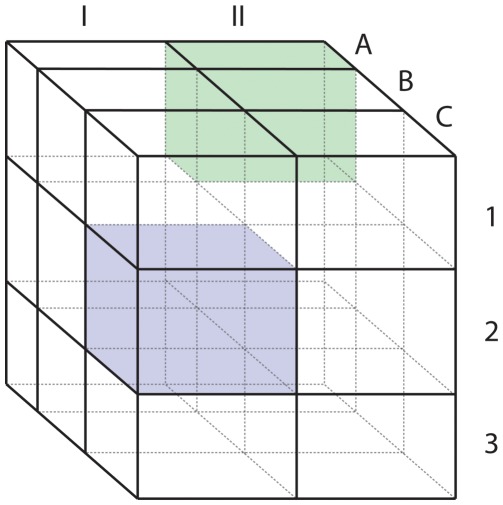
A three-dimensional representation of a paradigmatic classification of three dimensions (upper case letters, Roman numerals, and Arabic numerals). For example, any item possessing the attributes “I”, “C”, and “2″ would fall into the blue square class, while any item possessing the attributes “II”, A”, and “1″ would fall into the green square class. Redrawn and modified from (Figure 4 in [36]: 72).

A number of criticisms have been persuasively leveled against the standard practice of stone tool classification (called typology), however, including its subjective, non-quantitative nature ([33]) and the unavoidable inter-observer variability that it yields ([34];[35]). Our purpose here is not to further criticize subjective approaches to classifying stone tools, but to contrast them with an objective, logical alternative: paradigmatic classification. Dunnell ([36]) defined paradigmatic classification as a dimensional classification procedure in which the classes are defined by intersection, with each dimension being a set of mutually exclusive alternative features. However, all features belonging to a single dimension share the ability to combine with attributes of each other dimension. Dunnell ([36]) specified, “In paradigmatic classification all of the class definitions are drawn from the same set of dimensions of features. Individual classes are distinguished from one another by the unique product obtained in the combination, permutation, or intersection of features from the set of dimensions.” Figure 1 provides a visual representation of paradigmatic classification (see also [37];[38]).

Significantly, because a paradigmatic classification is produced by the intersection of dimensions of features, the maximum of classes possible for the assemblage under examination is fixed (see Figure 1 and caption), given the classification. In other words, the upper bound of richness is fixed and known a priori. In terms of estimating assemblage richness from a sample, this constraint is a fundamentally different one from what ecologists or biologists usually face because biological and ecological taxa are usually “extensionally” defined ([36]: 15). An extensional unit is derived by enumerating selected attributes shared by the unit’s members; the criteria comprising the unit are based on observed attributes of the actual members already placed in the unit. The characteristics of extensionally derived units are not theoretically informed in an explicit manner. As Dunnell ([36]: 15) notes, extensionally defined units are restricted in their utility to defining what is already known, i.e., extensional units are dependent on the specimens examined. Dunnell ([36]: 15) used the following example:

To define the term “dog” extensionally requires that you already know what dogs are in order to make the definitional listing. Ultimately, then, an extensional definition of a term simply means that something is that something because it is, and nothing more.

Alternatively, “intensionally” defined units, such as those created by paradigmatic classification, “specify a set of features which objects, whether known or unknown, must display in order to be considered referents for a given term” ([36]: 16). An intensional definition comprises the necessary and sufficient conditions for membership in a unit; it explicitly lists the distinctive attributes that a phenomenon must display to be identified as a member of the unit. The definitive attributes of the unit are derived from theory; there is no necessary reference to real, empirical specimens when the unit is constructed. The fact that something might not exist has no bearing on unit construction.

### An Example of Incompatibility

In a study by Eren ([39]), a non-parametric estimator, Chao1 ([14]), was used to estimate richness of paradigmatic classes of stone tools from seven late Pleistocene archaeological sites in the Lower Great Lakes region of North America. Here, “non-parametric” means that we do not need to specify a class abundance distribution. Thus a non-parametric estimator can be applied to all types of class distributions. The Chao1 estimator, developed for ecological applications, is based on the concept that rare species carry the most information about the number of species present in the assemblage, but not observed in a sample from it. Thus Chao1 uses only the singletons (species represented in the sample by only one individual) and doubletons (species represented in the sample by exactly two individuals) to estimate the number of unobserved species ([2];[14]). Importantly, a 95% confidence interval can be calculated for this richness estimator ([40]). (See Section 3 for details and formulas for the Chao1 estimator and its associated confidence interval.).

The stone tools under analysis are known as “unifacial stone tools,” a family of tools used by Clovis Paleoindians in Late Pleistocene North America (≈11,570 – 10,800 BP, [41∶254]) for a variety of scraping, cutting, and engraving tasks (for examples, see Figures S1, S2, S3, S4, S5). Criteria for two paradigmatic classifications were devised to classify, first, the overall shape of a stone tool and, second, the shape of its constituent parts (its edges). An analogous situation would be the creation of two classification schemes for, first, the shape of Swiss Army knives and, second, the gadgets contained within each one. The “tool shape” paradigmatic classification consisted of three dimensions with three, six, and six, features, respectively, for a total of 108 possible classes (3*6*6 = 108). The “edge shape” paradigmatic classification included four dimensions, with four, three, three, and three features, respectively, also for a total of 108 possible classes (4*3*3*3 = 108). (For details on the dimensions and features of the paradigmatic classifications used here, see the Materials S1 and Figures S6, S7, S8, S9, S10, S11, S12, S13, S14, S15, S16.).

When the Chao1 estimator was used to estimate paradigmatic class richness, an impossible estimate emerged: the upper 95% confidence interval of class richness sometimes exceeded the maximum number of possible classes (Tables 1 and 2, column 8) This discrepancy indicated to us that a new method was needed to address richness estimation when both upper and lower bounds are known. We introduce here doubly-bounded confidence intervals (both lower and upper bounds fixed) for class richness.

**Table 1:** The Chao1 estimate for tool class data, its standard error, and the 95% confidence interval for each of the seven sites (see Section 3 for notation and formulas).

Site	n	S_obs_	Singletons	Doubletons	Chao1 estimate	Standard error	95% confidence interval
Arc	134	31	12	7	41.28	7.78	33.74 – 69.48
Butler	63	23	9	6	29.75	5.88	24.54 – 52.44
Gainey	31	23	16	6	44.33	14.52	29.36 – 94.51
Leavitt	33	20	14	2	69.00	43.99	30.85 – **241.14**
Paleo Crossing	159	25	8	4	33.00	7.48	26.69 – 62.84
Potts	41	20	10	3	36.66	14.84	32.72 – 94.54
Udora	97	31	17	6	56.08	16.03	39.34 – **110.96**

The last column, obtained from Eq. (4), shows that the upper limits of the 95% confidence intervals for Leavitt and Udora Sites (boldfaced) exceeded the maximum possible value of 108.

**Table 2:** The Chao1 estimate for edge class data, its standard error, and the 95% confidence interval for each of the seven sites (see Section 3 for notation and formulas).

Site	n	S_obs_	Singletons	Doubletons	Chao1 estimate	Standard error	95% confidence interval
Arc	834	36	14	2	85.99	43.99	46.85 – **257.14**
Butler	272	24	10	1	74.00	59.58	31.95 – **339.63**
Gainey	203	25	8	4	33.00	7.48	26.69 – 62.84
Leavitt	222	26	9	1	66.50	48.08	32.28 – **287.05**
Paleo Crossing	1220	43	16	3	85.66	33.23	54.06 – **207.54**
Potts	351	25	5	2	31.25	7.55	25.97 – 65.13
Udora	634	37	15	3	74.50	29.68	46.55 – **184.15**

The last column, obtained from Eq. (4), shows that the upper limits of the 95% confidence interval of all sites except for Gainey and Potts (boldfaced) exceed the maximum possible value of 108.

## Methods

### Chao 1 Estimator

In this paper, we specifically illustrate our new method using the Chao1 estimator ([14]), although any estimator of class richness can be used in our method. Let S be the true unknown class richness of the assemblage and let S_obs_ be the number of observed classes in an empirical sample of size n from the assemblage, which we call the reference sample. We assume the fixed maximum for S is U (in our archaeological example, U = 108, as described earlier).

If an assemblage includes a non-negligible proportion of rare classes that may remain undetected in a sample of limited size, then the observed richness in the sample is likely to substantially underestimate the true richness. The abundant classes, which are virtually certain to be detected in samples, contain almost no information about the undetected classes, whereas rare classes, which are likely to be either undetected or infrequently detected, contain almost all the information about the number of undetected classes. We define the abundance frequency count f_k_ as the number of classes each represented by exactly k artifacts in the reference sample, 0≤ *k* ≤ *n*_._ The number of classes present in the assemblage but not detected in the reference sample is thus represented as f_0_.

The Chao1 estimator uses only the number of singletons (f_1_) and doubletons (f_2_) and the observed richness to obtain the following estimator for the class richness ([14]):
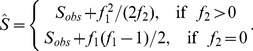
(1)


with an associated variance estimator of (if *f*_2_>0):
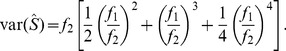
(2)


If *f*_2_ = 0, the variance formula (2) becomes:
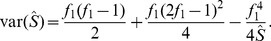
(2a)


Chao et al. ([42]) showed that, under many class abundance distributions, the Chao1 estimator, originally derived as an estimate of minimum possible richness, is very sharp if the reference sample size is large enough. This justifies the use of the Chao1 estimator as a valid estimator for large n. Since sampling variation is unavoidable, a confidence interval, which indicates the possible range of class richness based on the Chao1 estimator, should be reported to reflect sampling uncertainty. From a statistical point of view, the information about a fixed maximum does not help find a more accurate nonparametric point estimator for class richness, but it can be incorporated into the construction of a confidence interval such that the upper limit of the resulting interval is at most the maximum value U.

Bootstrapping is an approximation method that is widely used to assess sampling variability and to obtain confidence intervals for complicated estimators ([43];[44]). If we were to regard the reference sample of n artifacts that we collected as an “assemblage” and generate a series of bootstrap samples by randomly selecting n artifacts, with replacement, from the reference sample, we could calculate a Chao1 estimate of class richness, called a bootstrap estimate S*. Repeating this resampling procedure many times would produce many bootstrap estimates, forming a distribution that could be used for statistical inference in estimating at confidence interval.

In fact, we do not need to do bootstrap resampling because the bootstrap idea suggests an analytic method to obtain a confidence interval when both minimum and maximum bounds on the true class richness are known. We first review the method to construct a singly-bounded confidence interval with the lower bound no less than the observed richness. In most applications, the distribution for the undetected number of classes is right skewed, thus it is reasonable to assume a log-normal distribution for the number of undetected classes. Thus, we can assume that Y = log(S^*^ − S_obs_) is a normal distribution with mean 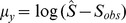 and variance σ^2^. It follows from the properties of a log-normal distribution that.
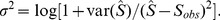
(3)


Then a 95% confidence interval for class richness is ([41]).
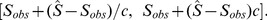
(4)where



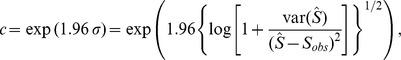
and 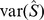 is given in Eq. (2). The lower limit of the resulting confidence interval is not lower than the observed class richness. In the last column of Tables 1 and 2, we show the confidence interval computed from Eq. (4) for each site. However, as explained earlier, some of the upper limits (boldfaced entries in the tables) exceed the maximum value of 108.

Here we propose a new analytic method based on the bootstrap idea to incorporate the maximum value U in the construction of confidence intervals, yielding a doubly-bounded confidence interval. Since any sensible estimate S^*^ should satisfy S_obs_ ≤ S^*^ ≤ U, equivalently, all reasonable values of Y = log(S^*^ − S_obs_) should be less or equal to V = log(U − S_obs_). Therefore, instead of the usual normal distribution, the distribution of Y follows a “truncated” distribution with the following density function (here “truncated” means that we only consider those Y values less than or equal to V.).
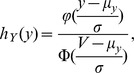
where φ and Φ denote, respectively, the probability density function and cumulative distribution function of the standard normal distribution. Let 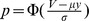, then a 1−α confidence interval for log(S − S_obs_) is



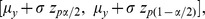
where z_α_ is a lower percentile point of a standard normal distribution, i.e., Φ(z_α_) = α and σ is defined in Eq. (3). As a result, the 1− α confidence interval for S is



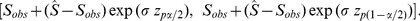
(5)The intervals in Equations (4) and (5) are both non-symmetric with respect to the richness estimate due to the log-transformation.

In the online Supporting Information (see Appendix S1 and Table S1 (spreadsheet)), using the edge class data for the Udora site (Table 2), we provide full calculation details to illustrate how to compute the new, doubly-bounded confidence interval. The traditional (singly-bounded) interval, Eq. (4), yields a 95% confidence interval of (46.55, 184.15) for which the upper limit exceeds 108. The new method, Eq. (5), yields a 95% confidence interval of (46.02, 104.36). Hence this example shows that the lower limit of the new interval is at least the observed class richness, while, simultaneously, the upper limit is less than 108. The doubly-bounded confidence interval for each site is shown in Tables 3 and 4.

**Table 3:** Comparison of traditional (singly-bounded) and new (doubly-bounded) confidence intervals for tool class data (the doubly-bounded interval is obtained from Eq. 5).

Site	Traditional 95% confidence interval	New 95% confidence interval
Arc	33.74 – 69.48	33.75 – 68.90
Butler	24.54 – 52.44	24.55 – 52.31
Gainey	29.36 – 94.51	29.34 – 87.14
Leavitt	30.85 – 241.14	30.01 – 103.80
Paleo Crossing	26.69 – 62.84	26.69 – 62.08
Potts	32.72 – 94.54	23.71 – 83.88
Udora	39.34 – 110.96	39.29 – 96.05

**Table 4:** Comparison of traditional (singly-bounded) and new (doubly-bounded) confidence intervals for edge class data (the doubly-bounded interval is obtained from Eq. 5).

Site	Traditional 95% confidence interval	New 95% confidence interval
Arc	46.85 – 257.14	45.65 – 105.36
Butler	31.95 – 339.63	30.92 – 104.06
Gainey	26.69 – 62.84	26.69 – 62.08
Leavitt	32.28 – 287.05	31.66 – 103.29
Paleo Crossing	54.06 – 207.54	53.11 – 105.62
Potts	25.97 – 65.13	25.97 – 63.24
Udora	46.55 – 184.15	46.02 – 104.36

### Interpolation (Rarefaction) and Extrapolation

Species richness estimators aim to estimate an asymptotic value, approached as the sample size tends to infinity. Colwell et al. ([3]) recently linked interpolation and extrapolation curves as a smooth curve. This curve provides useful information on comparing species richness for finite sample sizes. The goal of rarefaction is to estimate the expected number of classes S(m) in a random set of m individuals from the reference sample (m < n). Suppose the observed class abundance for the ith class is denoted by X_i_. Then a minimum variance unbiased estimator (Smith and Grassle 1977) for S(m) is.
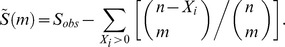



Colwell et al. ([3]) obtained an approximate unconditional variance estimator 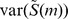 of the rarefied richness 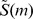. A traditional, symmetric 95% confidence interval is constructed by using 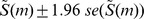.

The goal of extrapolation is to estimate the expected number of classes S(n+m*) in an augmented sample of n + m* individuals from the assemblage (m* >0). Shen et al. ([46]) derived the following estimator of S(n+m*):
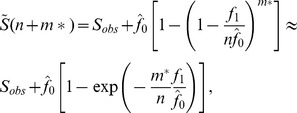
(6)where 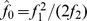
_,_ based on the Chao1 estimator. A variance estimator 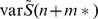 was also derived by Shen et al. ([45]). A symmetric 95% confidence interval for extrapolation is constructed as 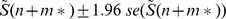.

**Figure 2:**
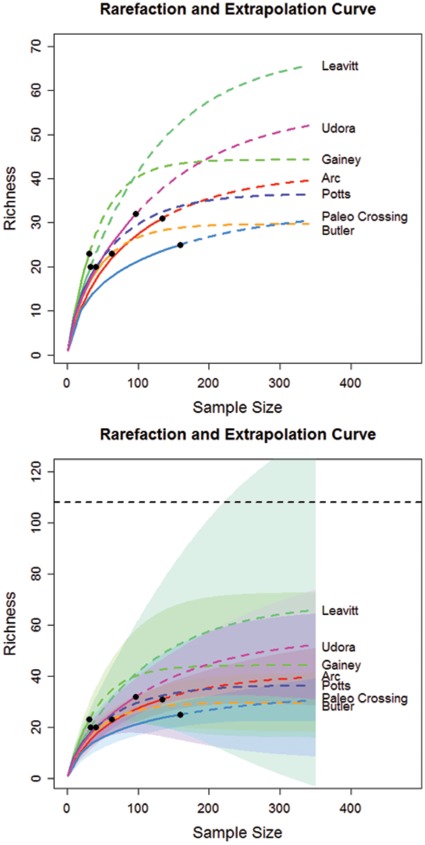
Rarefaction and extrapolation curves (upper panel) for tool class data from seven sites, with symmetric 95% confidence intervals (lower panel) based on Colwell et al. [3]. Black dots: the reference (empirical) samples. Solid lines: rarefaction curves. Dashed lines: extrapolation curves. Shaded area for each solid line: 95% confidence interval for the expected rarefied class richness. Shaded area for each dashed line: 95% confidence interval for the expected extrapolated class richness up to a sample size of 350.

**Figure 3:**
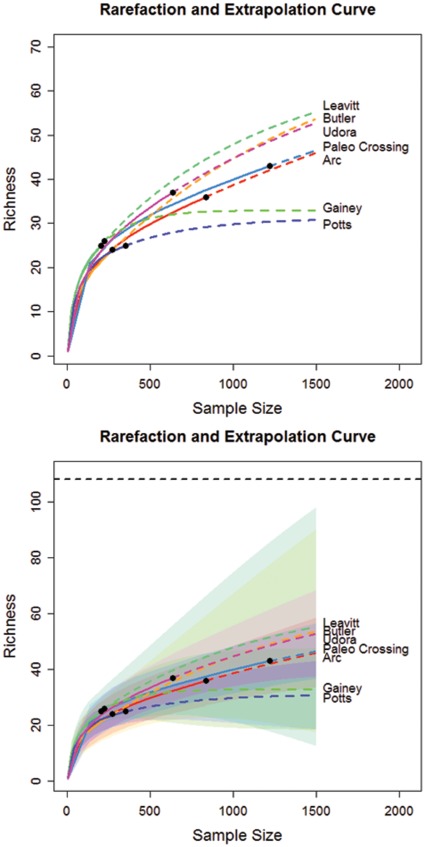
Rarefaction and extrapolation curve (upper panel) of seven sites for edge class data with symmetric 95% confidence intervals (lower panel) based on Colwell et al. [3]. Black dots: the reference (empirical) samples. Solid lines: rarefaction curves. Dashed lines: extrapolation curves. Shaded area for each solid line: 95% confidence interval for the expected rarefied class richness. Shaded area for each dashed line: 95% confidence interval for the expected extrapolated class richness up to a sample size of 1500.



In Figure 2, we show the plots of rarefaction and extrapolation for tool class data from seven sites. The corresponding plots for edge class data are shown in Figure 3. In Figure 2, the upper limit of the traditional symmetric 95% confidence interval of the predicted class richness for the Leavitt Site is greater than the maximum value of 108 when sample size exceeds 200. We now briefly describe the modifications required for the confidence interval of the extrapolation part of the curve, when when there is a fixed maximum value for class richness. If we assume that the logarithm of bootstrap estimates of S(n+m*) is a normal distribution truncated by log(U), then a parallel derivation to that in Section 3 for obtaining Equation (5) yields a 1− α confidence interval for S(n+m*) given by.

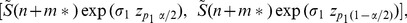
(7)where



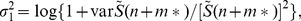
(7a)and we define p_1_ as 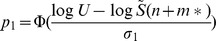. A similar approach can be also applied to the rarefaction part of the curve simply by replacing 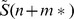 and its variance by 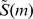 and its variance. Thus, the 1− α confidence interval for S(m) is



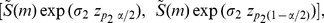
(8)where



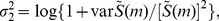
(8a)and p_2_ is defined as 
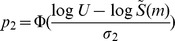
.

**Figure 4:**
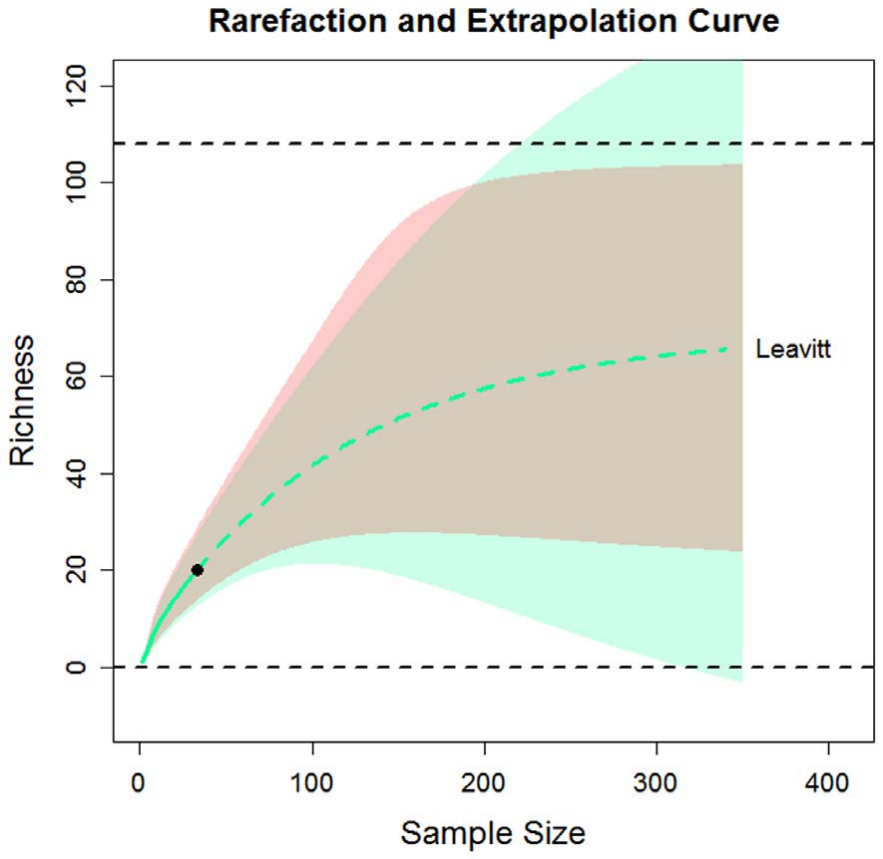
Comparison of the symmetric intervals (wider intervals, as in Figure 2) and the doubly-bounded confidence interval for tool class data from Leavitt Site. The symmetric intervals were obtained based on Colwell et al. [3] and the doubly-bounded intervals were computed from Equations (7) and (8). The intervals unavoidably tend to be wide due to the small sample size (n = 33) for the site. Long-range extrapolation is applied only to illustrate the behavior or the bounded confidence interval.

**Figure 5:**
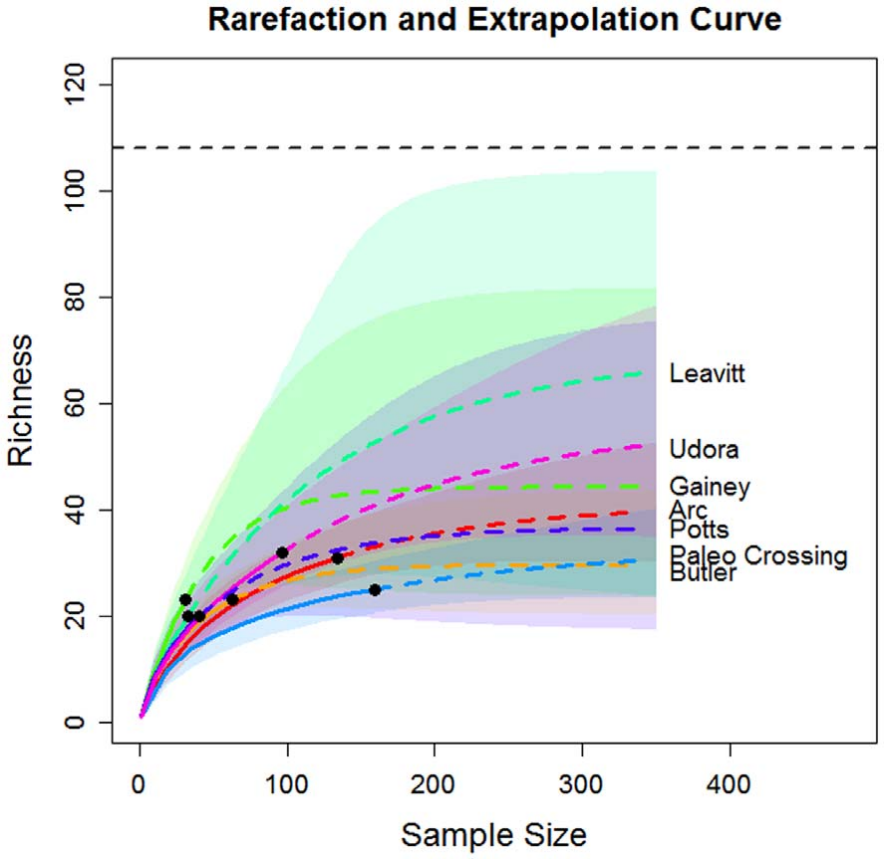
Rarefaction and extrapolation curves for tool class data from seven sites with doubly-bounded 95% confidence intervals based on Equations (7) and (8). Black dots: reference samples. Solid lines: rarefaction curves. Dashed lines: extrapolation curves. Shaded area for each solid line: 95% confidence interval for the expected rarefied class richness. Shaded area for each dashed line: 95% confidence interval for the expected extrapolated class richness up to a sample size of 350.

**Figure 6:**
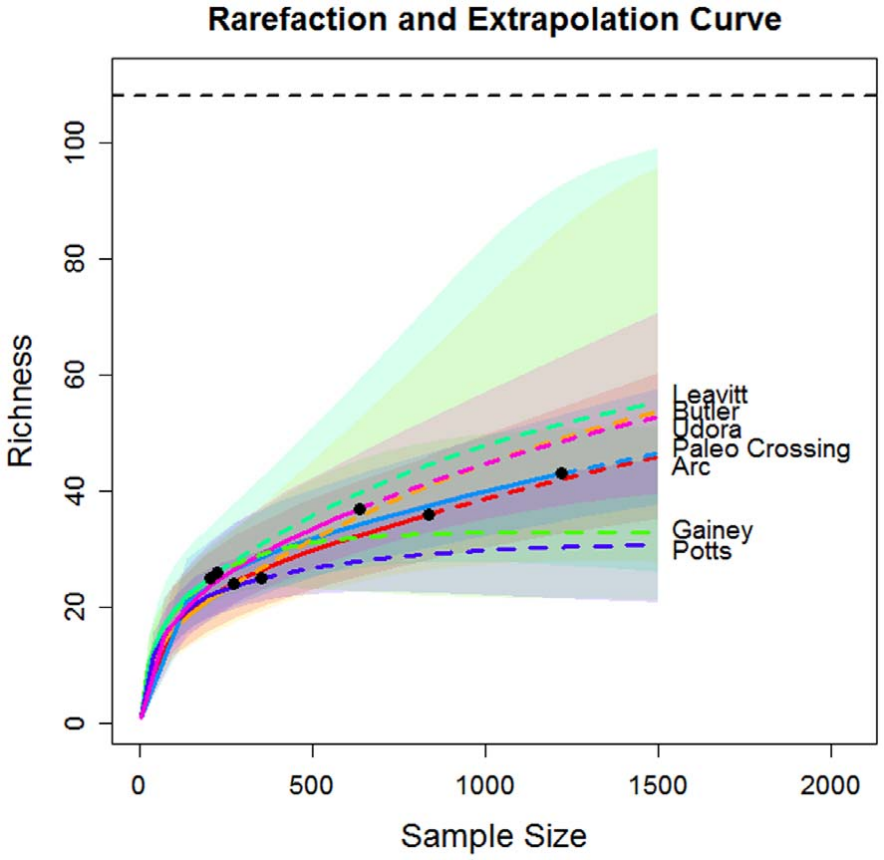
Rarefaction and extrapolation curves for edge class data from seven sites with doubly-bounded 95% confidence intervals based on Equations (7) and (8). Black dots: reference samples. Solid lines: rarefaction curves. Dashed lines: extrapolation curves. Shaded area for each solid line: 95% confidence interval for the expected rarefied class richness. Shaded area for each dashed line: 95% confidence interval for the expected extrapolated class richness up to a sample size of 1500.

In Figure 4, we single out the Leavitt Site to compare the original symmetric and the modified confidence intervals. The sample size for tool class in Leavitt Site is only 33, thus the variance of the Chao1 estimator is the largest of the seven sites. When we extrapolate to 350, it is unavoidable that the confidence intervals become wide. The comparison of seven sites with the modified confidence intervals are shown in Figure 5 for tool class data and in Figure 6 for edge class data. It is clear that for any finite sample sizes, all seven intervals overlap substantially. Although slight overlap may not imply significance, the considerable overlap among these confidence intervals indicates that the current data do not support any significant difference in class richness, among the seven sites.

## Results and Discussion

Based on the work of Bettinger ([46]), Schiffer ([47]), and Surovell ([48]), Eren ([39]) proposed that different forager base camp settlement patterns would be corroborated by different levels of tool class and edge class richness, by the pattern of relative abundance among classes, and by the classes represented in artifact assemblages (see also [49];[50];[51];[52];[53];[54]). In regard to richness only, a residential forager settlement pattern (moving a base camp across the landscape short distances, but frequently, to complete different subsistence tasks) would be supported if the unifacial stone tool class and edge class richness differed significantly among the seven base camp sites. The rationale behind this inference is that a sample of base camp sites used by a group of foragers following a residential mobility strategy would be less likely to exhibit the same scope of tool-using activities (and thus tool class and edge class richness) at all sites, since each is positioned in a unique location across a landscape for a different subsistence purpose. Alternatively, a logistical forager settlement pattern (moving a base camp far across the landscape, but less often) would be supported if tool class and edge class richness varied little among sites. In a logistical mobility strategy, base camps are occupied for much longer periods, requiring relatively more subsistence tasks to eventually be completed at a single location. If so, a sample of logistical base camp sites is more likely to reveal similar spectra of tool-using activities (and thus tool and edge class richness), as the same wide scope of activities will be eventually carried out at each.

The original (singly-bounded) 95% confidence intervals of the seven base camp sites’ tool and edge class richness (Table 3 and 4, column 1, Figures 2 and 3) did not allow any inference about forager settlement patterns because they did not make any empirical or logical sense. However, with confidence intervals constrained by the maximum class richness (Table 3 and 4, column 1, Figures 5 and 6), it is now clear that the new 95% confidence intervals overlap substantially, suggesting that tool class and edge class richness do not vary significantly among the sites. Our conclusion is justified from both asymptotic richness estimation (Table 3 and 4) and rarefaction-extrapolation methodology (Figures 5 and 6). On its own this result supports the notion that Late Pleistocene Clovis foragers in the Lower Great Lakes used a base camp settlement pattern that was probably more logistical in nature than residential, though future assessments should consider this result among a suite of other diversity measures and archaeological evidence.

The applicability of our new method is not limited to archaeology or paradigmatic classification. Indeed, it can be applied to any set of data for which there is a fixed maximum number of classes:

### Site Occupancy Models

In site occupancy models ([55]), a fixed maximum number of U sites may either be occupied or unoccupied by a member of each class. The site occupancy rate can be estimated by S_est_/U, where S_est_ is interpreted as the estimated number of sites at which the class is present. Therefore, because an upper bound for any estimate is the number of sites, our method can be applied to site occupancy models. In the previous literature, the estimated upper limit of a confidence interval of the occupancy rate may exceed one because the estimate may exceed the number of sites. By contrast, the new method avoids this obvious impossibility.

### A Marketing Example

Suppose a manufacturer of athletic shoes has a current range of products that includes exactly U shoe styles. To efficiently target company advertising, the manufacturer’s marketing division wants to estimate the relative abundance and the total number of the company’s shoe styles currently worn on university campuses in different regions of several countries. Because students may well have purchased shoes far from the campus, even in a different country, for students at highly international universities, local sales data from shops near campuses are not reliable.

Instead, the marketing department hires local observers at each campus to count the number of students they observe over a specified period wearing each of the U styles. The relative abundance of the styles recorded at each campus can be approximated, for these purposes, from the proportions observed, but the total number of styles actually worn on a campus may lie anywhere between the observed number (S_obs_) and U. Our new method (Equations 1 and 5) can provide an appropriate estimate with sensible confidence intervals.

### A Census Example

Suppose a social/political scientist is conducting research on the sociocultural richness (as measured by the number of distinct sociocultural groups represented) of people in geographic regions or neighborhoods where that information would be difficult to obtain by an exhaustive census, for practical or logistical reasons (war zones, hazardous terrain, cost of surveying an entire population). Characteristics such race, religion, nationality, or socio-economic status could be assessed from the sample of people who are most easily and/or safely accessible, from which a fixed number of sociocultural categories (classes, in the statistical sense) could be defined. The application of our estimators would allow for an assessment of true sociocultural richness for each place, based on limited sampling, that would not otherwise be practical.

## Supporting Information

Figure S1 **Unifacial stone tools from the site of Paleo Crossing, Ohio.**
(TIF)

Figure S2 **Unifacial stone tools from the site of Paleo Crossing, Ohio.**
(TIF)

Figure S3 **Unifacial stone tools from the site of Paleo Crossing, Ohio.**
(TIF)

Figure S4 **Handheld use of a unifacial stone tools.**
(TIF)

Figure S5 **A hafted unifacial stone tool.**
(TIF)

Figure S6 **Visual criterial for defininf a unifacial stone tool.**
(TIF)

Figure S7 **Collins (1999) triangular coordinate graph.**
(TIF)

Figure S8 **Measurement of unifacial stone tool length, width, and thickness.**
(TIF)

Figure S9 **Measurement of the “width category” and “thickness category.”**
(TIF)

Figure S10 **Schematic examples of unifacial stone tool morphological classes.**
(TIF)

Figure S11 **Examples of unifacial stone tool edge sections.**
(TIF)

Figure S12 **Edge angle measurements.**
(TIF)

Figure S13 **Edge shape measurements.**
(TIF)

Figure S14 **Unifacial stone tool edge notches.**
(TIF)

Figure S15 **Unifacial stone tool edge spurs.**
(TIF)

Figure S16 **Schematic examples of unifacial stone tool edge morphological classes.**
(TIF)

Materials S1 **Schematic examples of unifacial stone tool edge morphological classes.**
(DOC)

Appendix S1 **An illustrative example for calculating doubly-bound confidence intervals.**
(DOC)

Table S1 **A spreadsheet for calculating doubly-bound confidence intervals.**
(XLSX)
