# Supplementary figures and images for: Estimating the Richness of a Population When the Maximum Number of Classes Is Fixed: A Nonparametric Solution to an Archaeological Problem
Source: PLoS One. 2012 May 29;7(5):e34179. doi: 10.1371/journal.pone.0034179 (PMC3362599; doi:10.1371/journal.pone.0034179)

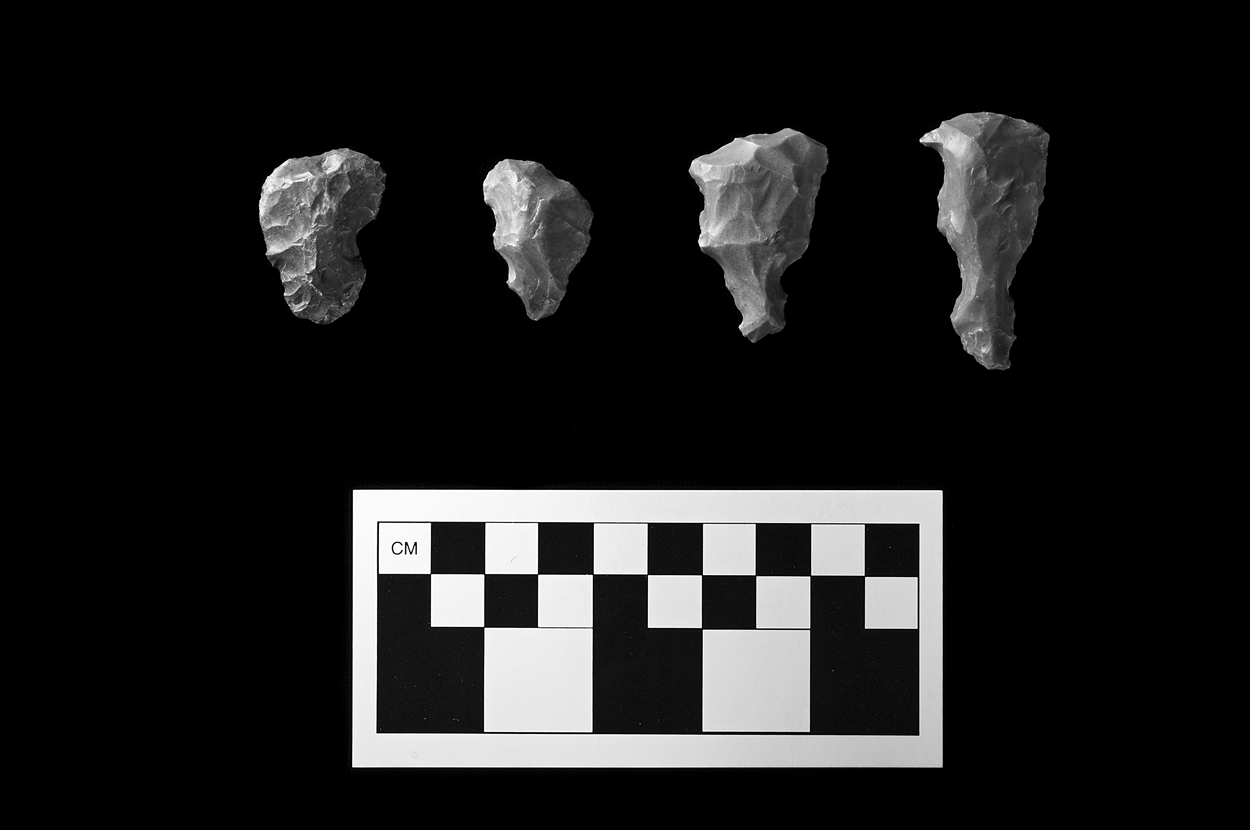

Supplement: Figure S1 — Unifacial stone tools from the site of Paleo Crossing, Ohio. (TIF) [file pone.0034179.s001.tif]

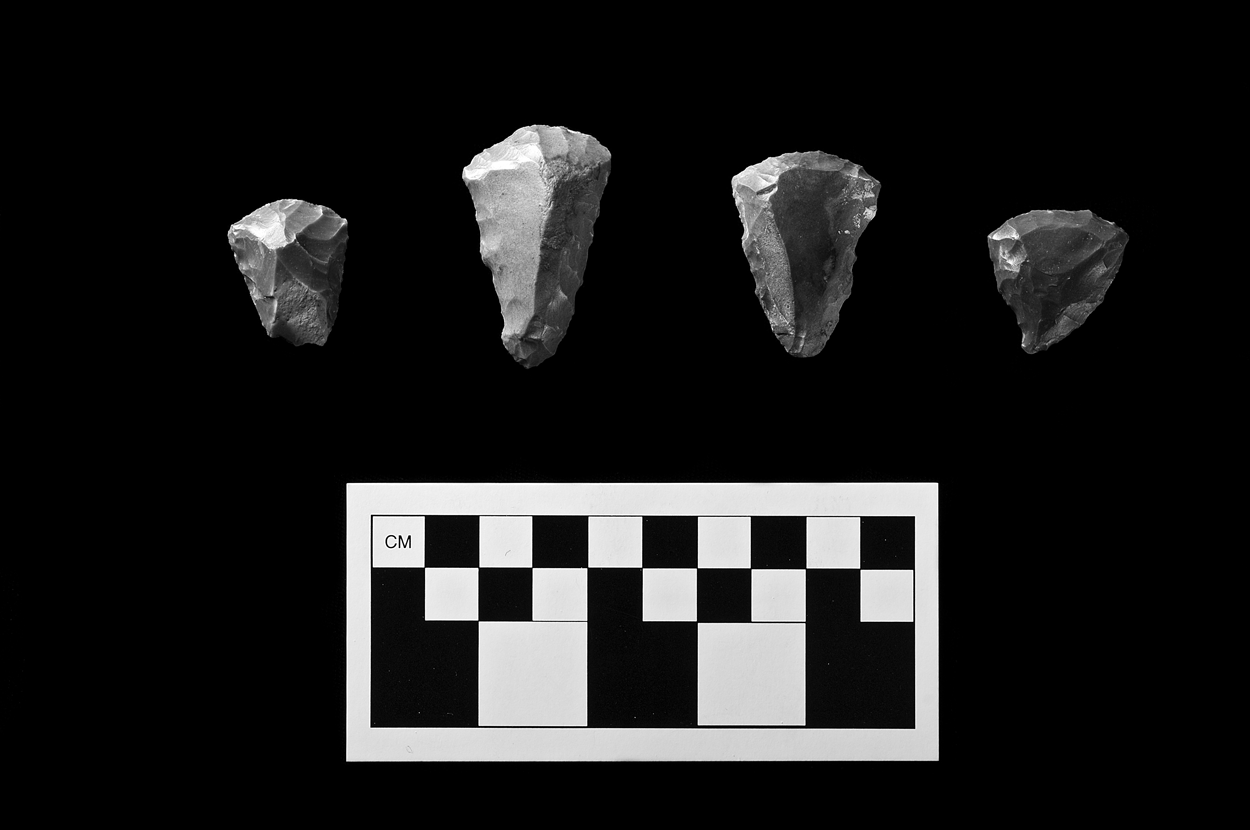

Supplement: Figure S2 — Unifacial stone tools from the site of Paleo Crossing, Ohio. (TIF) [file pone.0034179.s002.tif]

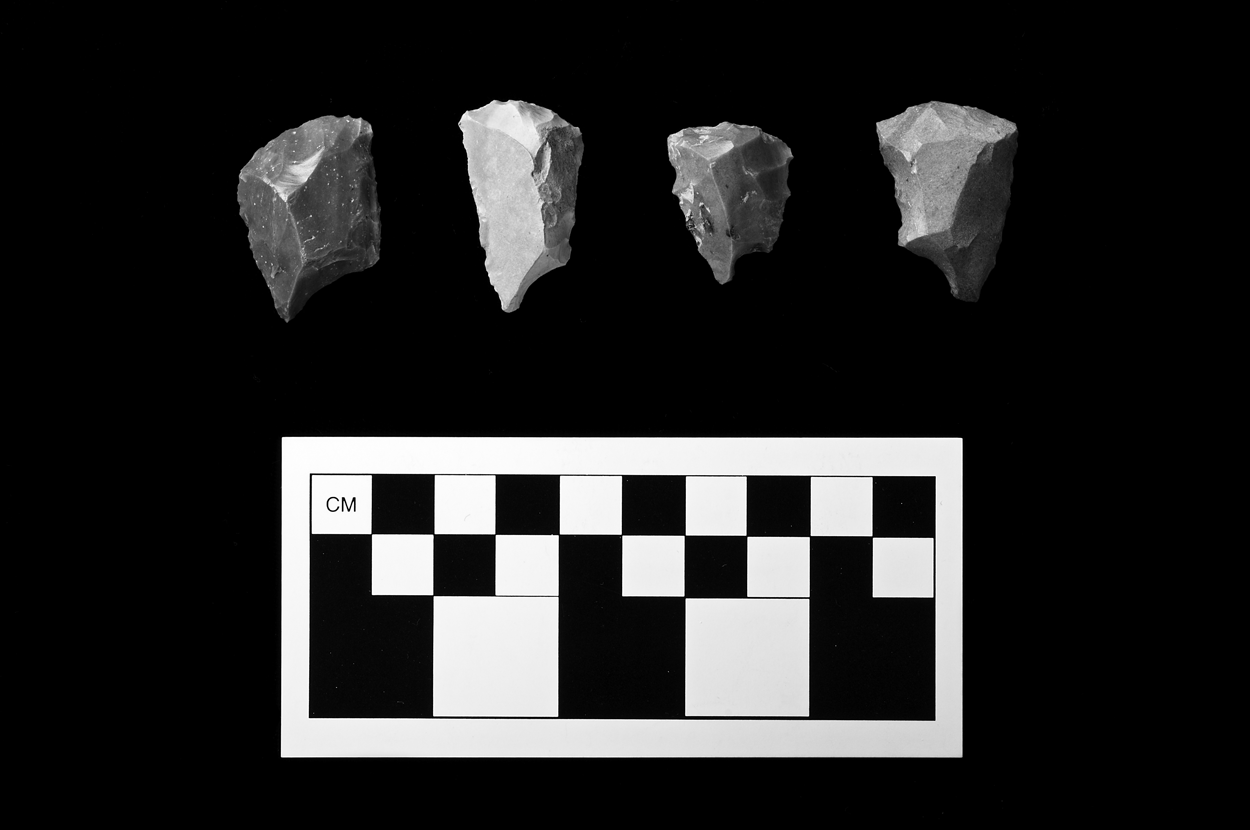

Supplement: Figure S3 — Unifacial stone tools from the site of Paleo Crossing, Ohio. (TIF) [file pone.0034179.s003.tif]

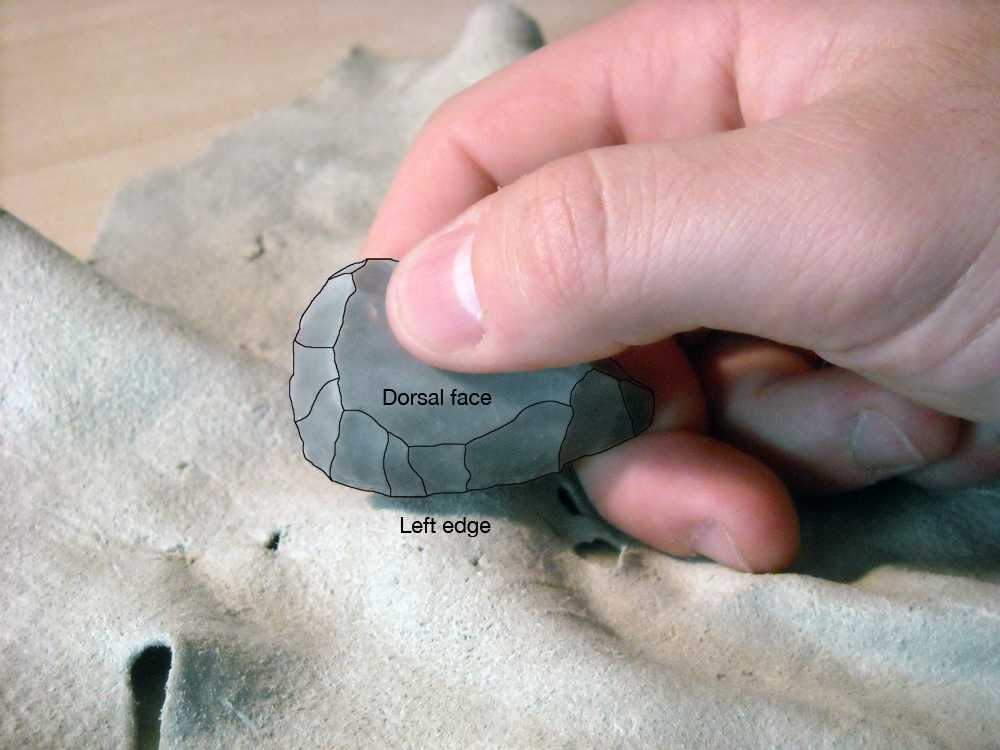

Supplement: Figure S4 — Handheld use of a unifacial stone tools. (TIF) [file pone.0034179.s004.tif]

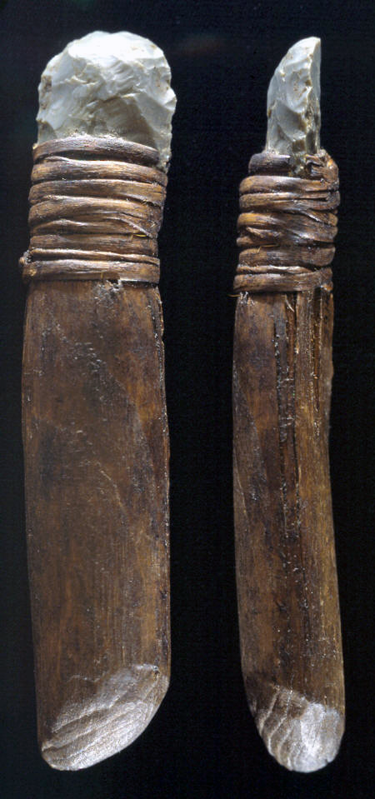

Supplement: Figure S5 — A hafted unifacial stone tool. (TIF) [file pone.0034179.s005.tif]

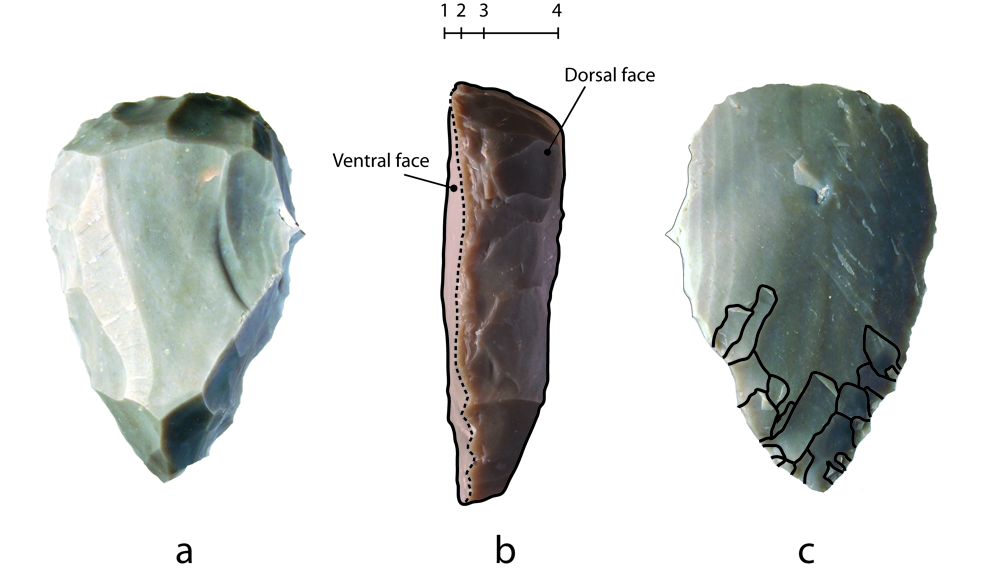

Supplement: Figure S6 — Visual criterial for defininf a unifacial stone tool. (TIF) [file pone.0034179.s006.tif]

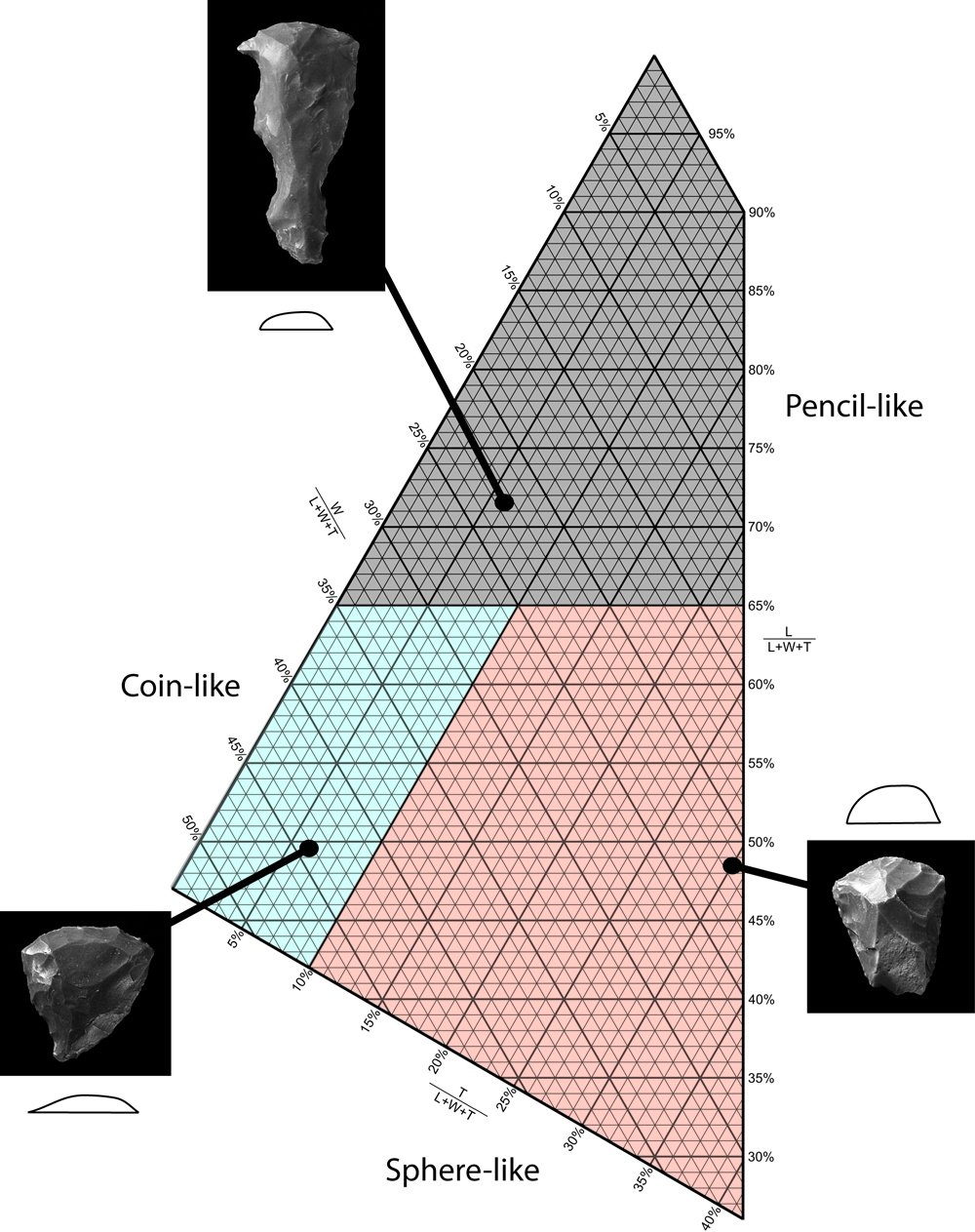

Supplement: Figure S7 — Collins (1999) triangular coordinate graph. (TIF) [file pone.0034179.s007.tif]

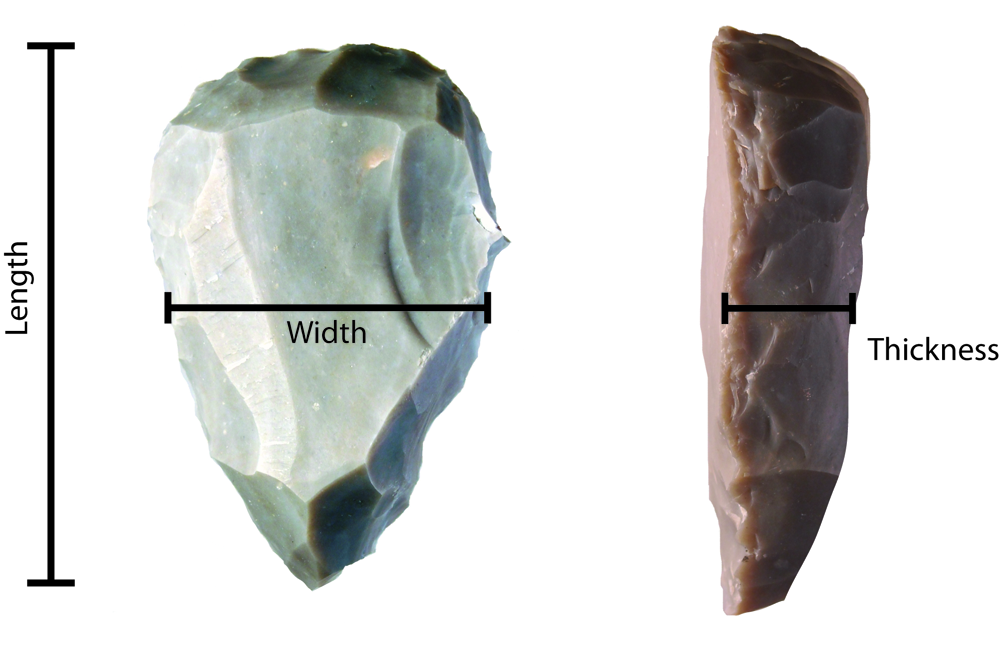

Supplement: Figure S8 — Measurement of unifacial stone tool length, width, and thickness. (TIF) [file pone.0034179.s008.tif]

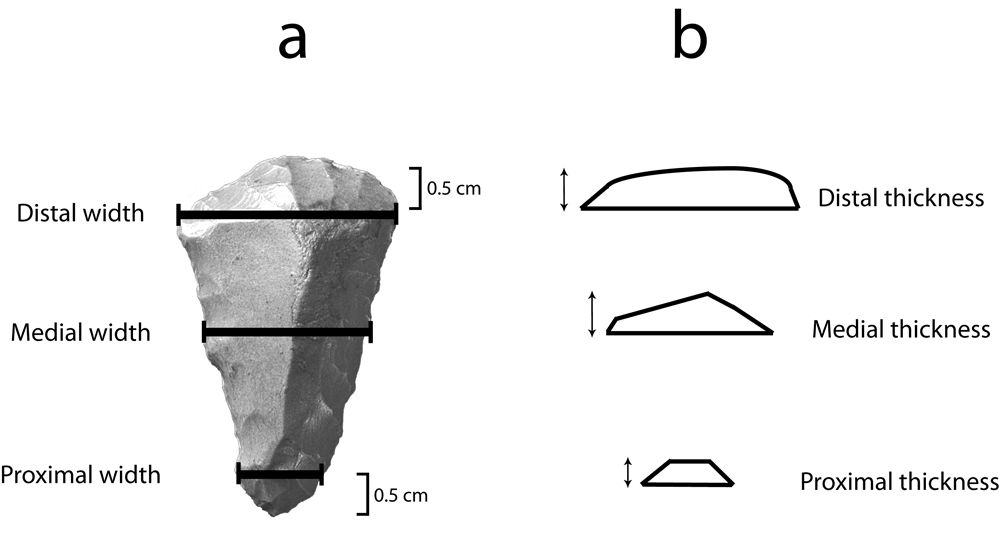

Supplement: Figure S9 — Measurement of the “width category” and “thickness category.” (TIF) [file pone.0034179.s009.tif]

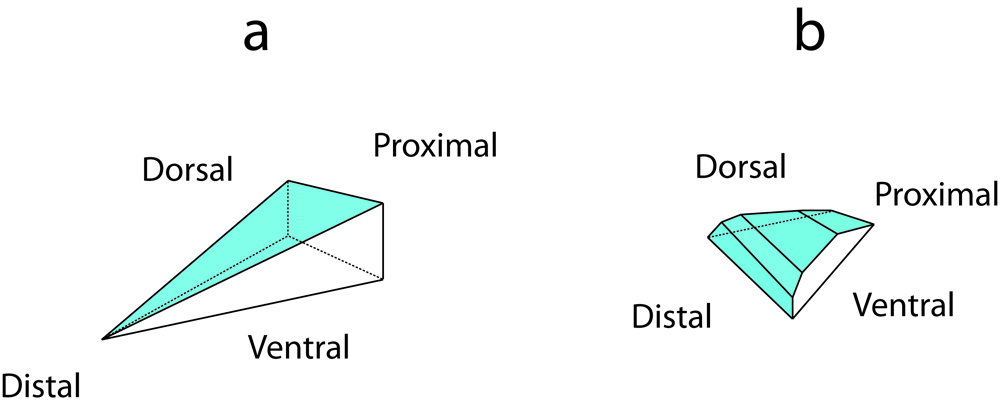

Supplement: Figure S10 — Schematic examples of unifacial stone tool morphological classes. (TIF) [file pone.0034179.s010.tif]

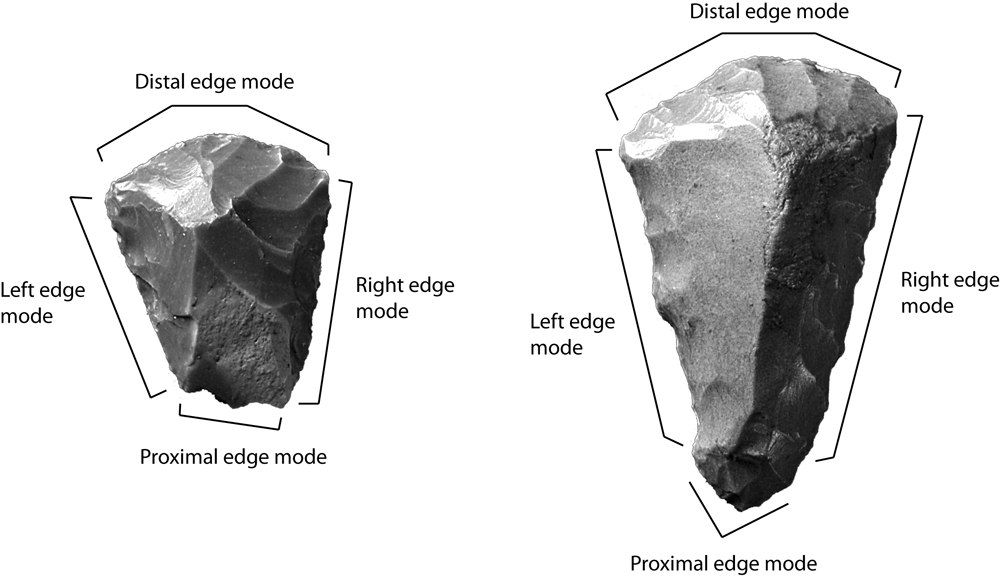

Supplement: Figure S11 — Examples of unifacial stone tool edge sections. (TIF) [file pone.0034179.s011.tif]

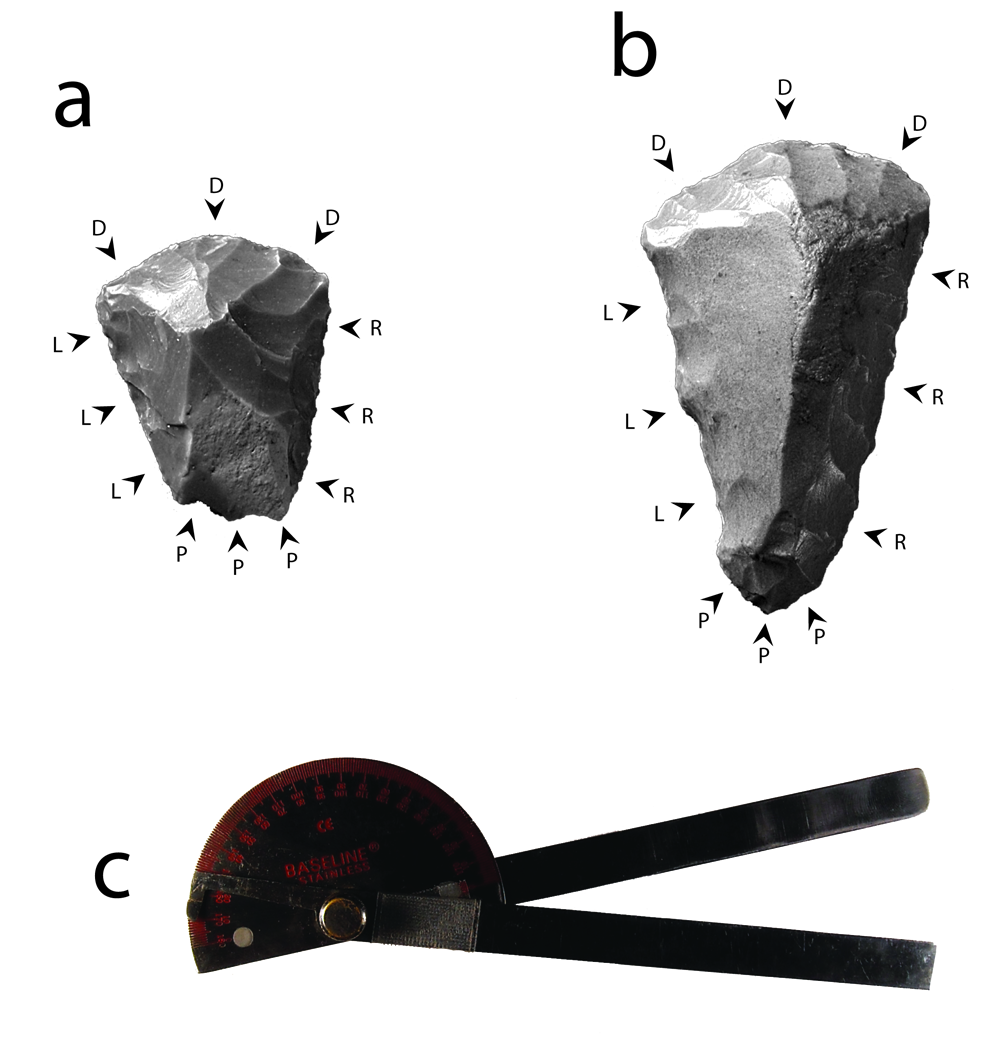

Supplement: Figure S12 — Edge angle measurements. (TIF) [file pone.0034179.s012.tif]

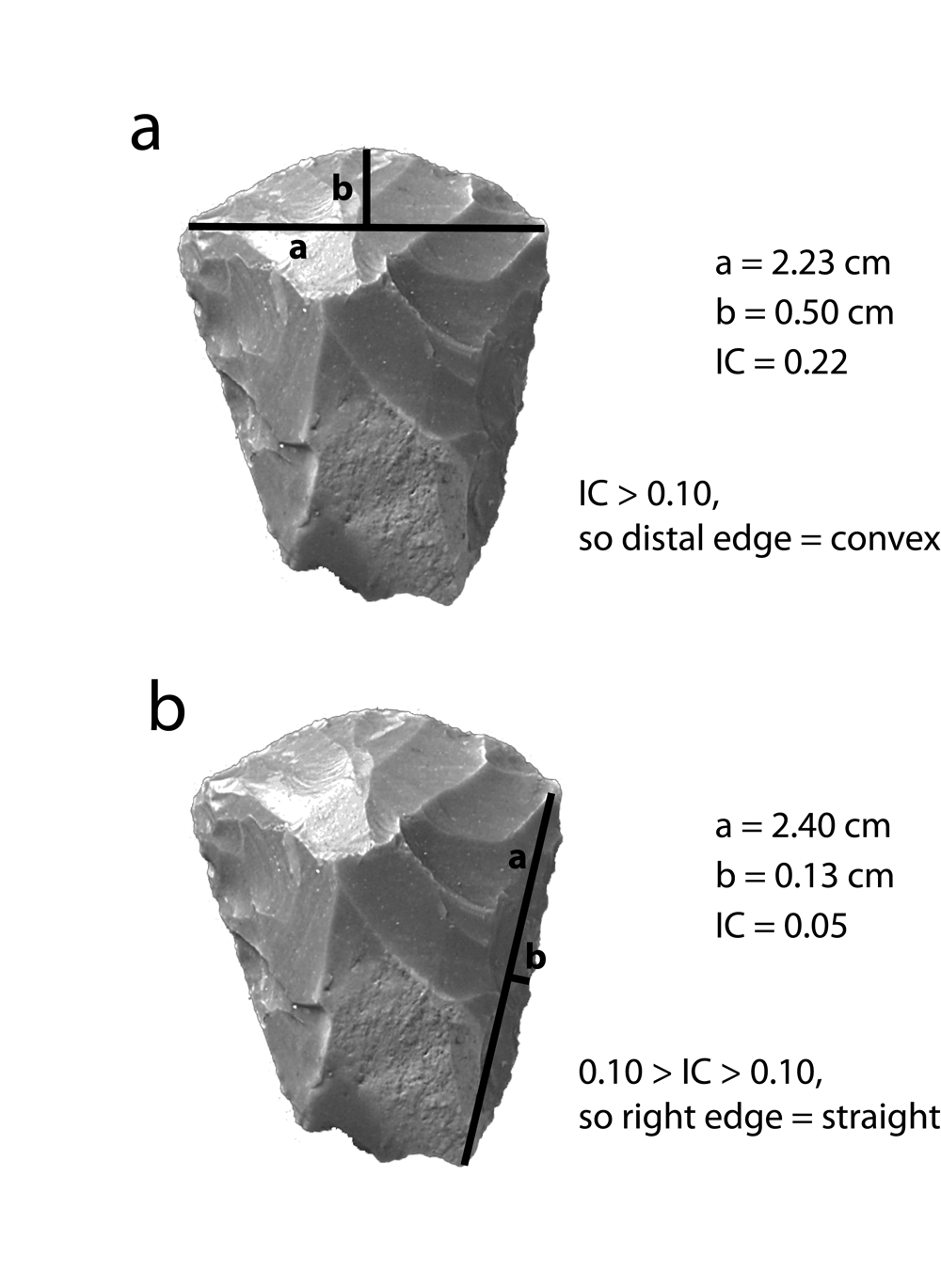

Supplement: Figure S13 — Edge shape measurements. (TIF) [file pone.0034179.s013.tif]

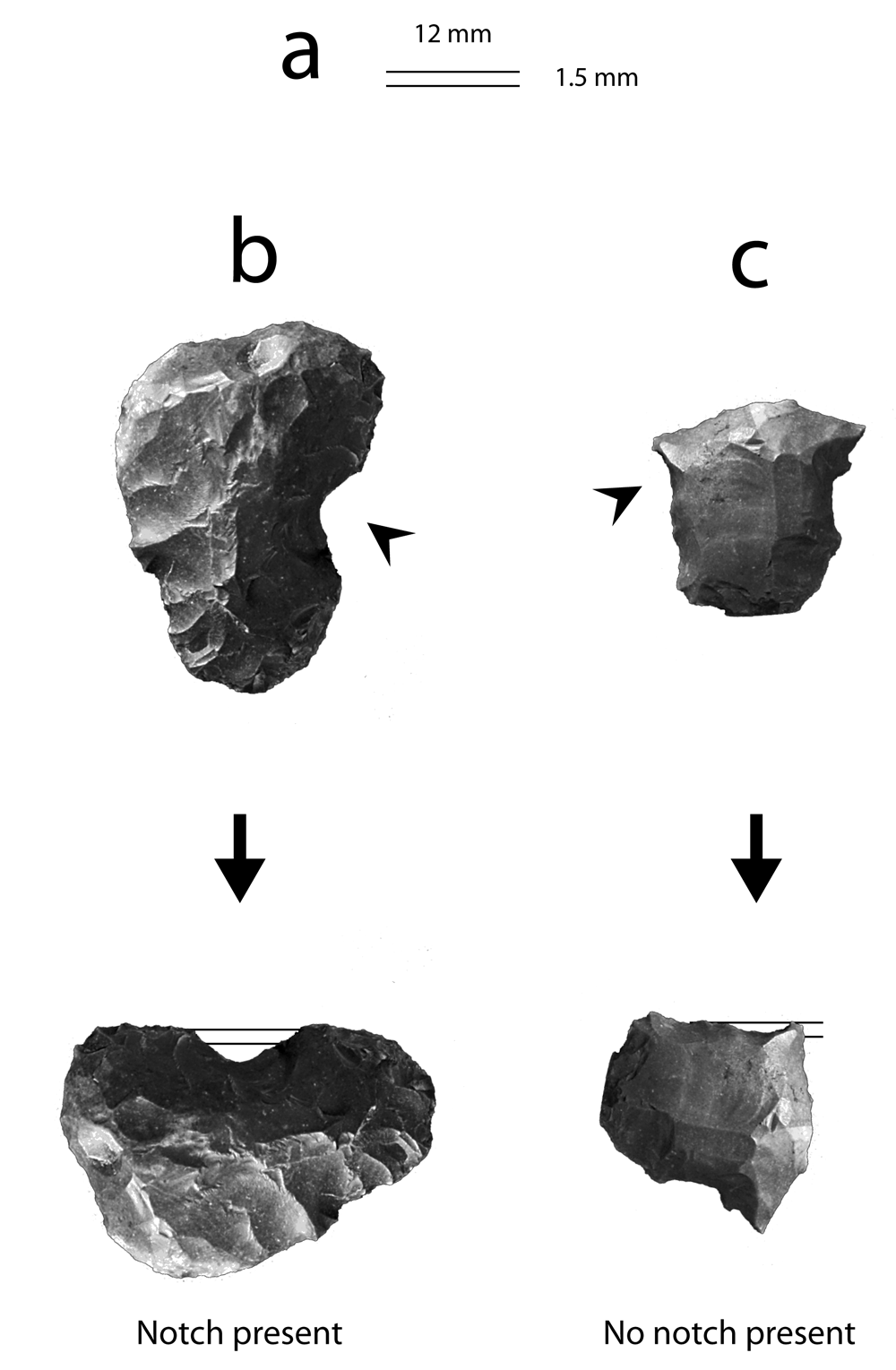

Supplement: Figure S14 — Unifacial stone tool edge notches. (TIF) [file pone.0034179.s014.tif]

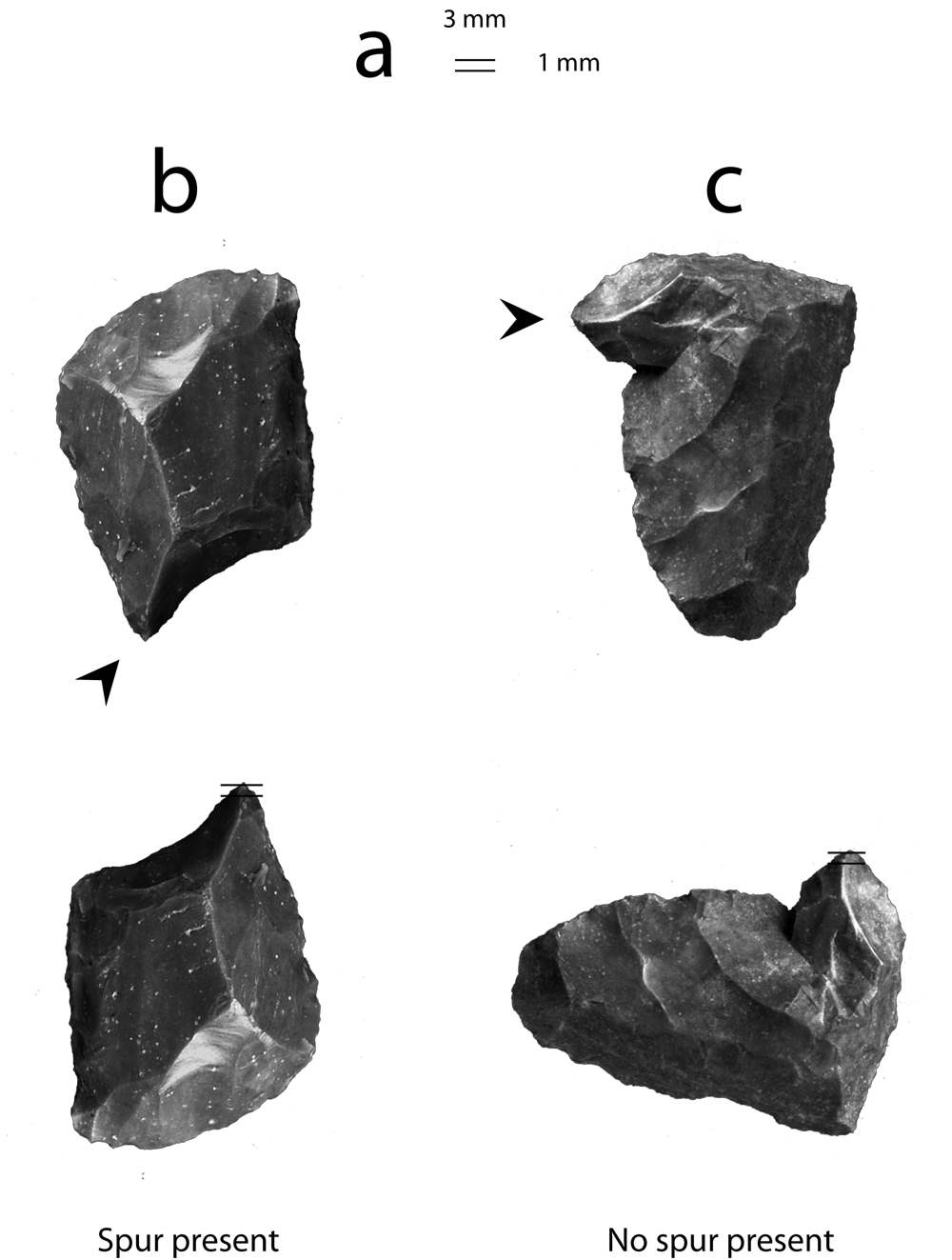

Supplement: Figure S15 — Unifacial stone tool edge spurs. (TIF) [file pone.0034179.s015.tif]

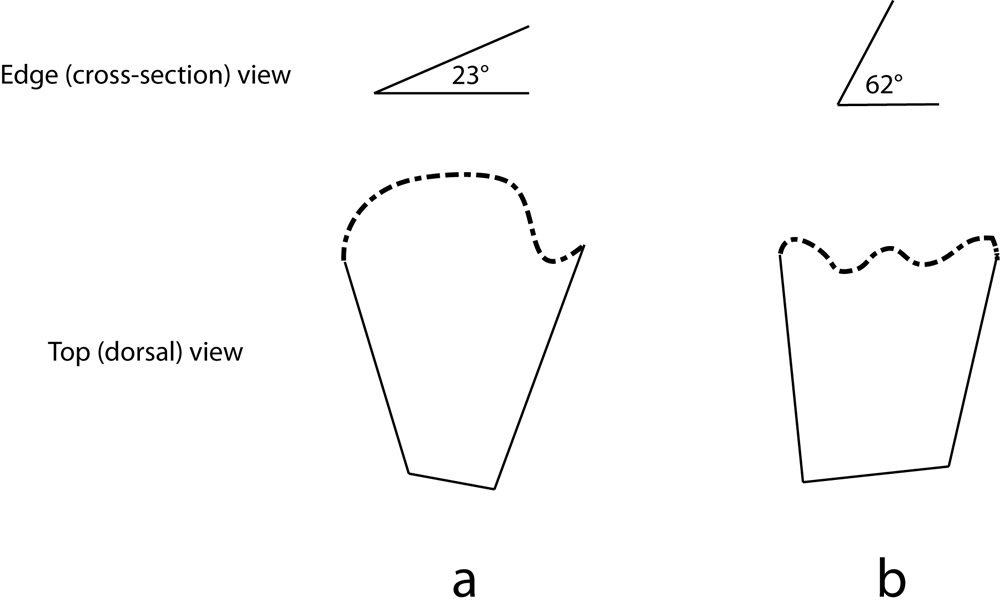

Supplement: Figure S16 — Schematic examples of unifacial stone tool edge morphological classes. (TIF) [file pone.0034179.s016.tif]
