# Supplementary material for: Estimating the Richness of a Population When the Maximum Number of Classes Is Fixed: A Nonparametric Solution to an Archaeological Problem
Source: PLoS One. 2012 May 29;7(5):e34179. doi: 10.1371/journal.pone.0034179 (PMC3362599; doi:10.1371/journal.pone.0034179)
Supplement: Materials S1 — Schematic examples of unifacial stone tool edge morphological classes. (DOC) [file pone.0034179.s017.doc]

Supplementary materials for:

Estimating the richness of a population when the maximum number of classes is fixed: a nonparametric solution to an archaeological problem

Metin I. Eren

Department of Anthropology, University of Kent, Canterbury, U.K.

Email: [metin.i.eren@gmail.com](mailto:metin.i.eren@gmail.com)

Anne Chao

Institute of Statistics, National Tsing Hua University, Hsin-Chu, Taiwan 30043

Email: [chao@stat.nthu.edu.tw](mailto:chao@stat.nthu.edu.tw)

Wenhan Hwang

Institute of Statistics, National Chung Hsing University, Taichung, Taiwan 402

Email: [wenhan@nchu.edu.tw](mailto:wenhan@nchu.edu.tw )

Robert K. Colwell

Department of Ecology and Evolutionary Biology, University of Connecticut, Storrs, Connecticut, U.S.A. 06269-3043

Email: [colwell@uconn.edu](mailto:colwell@uconn.edu)

S1. Examples of unifacial stone tools

[Figures S1-S5 here.]

S2. Stipulation of the field to be classified

Dunnell (1971: 52) states that the fundamental beginning to classification is “the subjective selection of the field for which the classification is to be constructed.” In other words, in order to classify unifacially flaked stone tools it is necessary to first define them externally to the classification itself. Here, a unifacially flaked stone tool is defined by three criteria (Figure S6): (1) Volumetrically, the edge where the ventral and dorsal faces of a flaked stone tool meet must be, on average, located no more than one third the distance of maximum tool thickness from the ventral face. (2) There must be retouch on the dorsal face of the tool (excluding utilized flakes). (3) In the event that there is retouch on the ventral face (e.g. bulb thinning flakes), the retouch must not cover more than 50% of the ventral face’s area.

[Figure S6 here]

S3. Details of the paradigmatic classification applied to the unifacial stone tools

[Figures S7-S16 here throughout]

Tool morphology describes the overall shape of a unifacially flaked stone tool, while edge morphology examines its four component sections (the proximal, distal, and left and right lateral edges). While the former reveals broad patterns in holistic tool design, the latter provides a finer look at each constituent tool element. Since each unifacially flaked stone tool can yield up to four edges (distal, proximal, right lateral, left lateral), obviously there will be a much larger sample size of edge specimens than tool specimens.

S3.1. The construction of tool morphological classes

Tool morphological classes are created by three dimensions, or “modes” (Dunnell 1971): (1) shape, (2) width category, and (3) thickness category.

S3.1.1. Mode 1: shape

The analysis of shape roughly follows the procedure detailed in Collins (1999), a fast but reliable method for plotting specimens in morphospace. Three ratios are calculated per tool:

The length ratio: Length / (Length + Width + Thickness)

The width ratio: Width / (Length + Width + Thickness)

The thickness ratio: Thickness / (Length + Width + Thickness)

Collins (1999) then plotted the three ratio values on a triangular coordinate graph to visually determine whether specimens were more “sphere-like,” “coin-like,” or “pencil-like” (Figure S7). Here, these three shape modes were defined quantitatively. A pencil-like specimen was any specimen with a length ratio greater than 0.65. A coin-like specimen was any specimen with a length ratio less than 0.65, but a thickness ratio less than 0.10. A sphere-like specimen was any specimen with a length ratio less than 0.65, but a thickness ratio equal to or greater than 0.10. These values were arbitrarily chosen based on the investigator’s perception of different shapes. A specimen identified as pencil-like was recorded as a “1”; coin-like as a “2”; and sphere like as a “3.”

Length was measured as the distance between the platform and the most distal point on the specimen parallel to the axis of percussion (Figure S8). Width was measured as the distance between the two lateral edges of the specimen at the mid-point of and perpendicular to the length measurement (Figure S8). Thickness was measured as as the distance between the dorsal and ventral faces of the specimen at the same location as the width measurement (Figure S8).

S3.1.2. Mode 2: width category

The width category mode identified the widest and narrowest parts of the specimen. Three width measurements were recorded for this mode: the proximal width, the distal width, and the medial width (Figure S9a). The proximal width was measured 0.5 cm from the most proximal point of the specimen, perpendicular to the axis of percussion. The distal width was measured 0.5 cm from the most distal point of the specimen, also perpendicular to the axis of percussion. The medial width is the same measurement as the width measurement presented in Supplementary Figure 8.

Once all three widths were measured they were ordered from least to greatest. For instance, a specimen with a proximal width of 1.40 cm, a medial width of 1.60 cm, and a distal width of 1.55 was categorized as “proximal-distal-medial” (“pdm”). Each specimen was assigned a number representative of each possible categorization:

Distal-medial-proximal (dmp) = 1 (distal is least wide, then medial, then proximal)

Distal-proximal-medial (dpm) = 2

Medial-distal-proximal (mdp) = 3

Medial-proximal-distal (mpd) = 4

Proximal-distal-medial (pdm) = 5

Proximal-medial-distal (pmd) = 6

In the event of a tie between two section widths, the following procedure was implemented:

(1) In the event of a tie between the proximal and medial section widths, the proximal was categorized as being less wide

(2) In the event of a tie between the proximal and distal section widths, the proximal section was categorized as being less wide.

(3) In the event of a tie between the medial and distal section widths, the medial section was categorized as being less wide.

S3.1.3. Mode 3: thickness category

The thickness category identified the thickest and thinnest parts of a specimen. Three thickness measurements were recorded for this mode: the proximal thickness, the distal thickness, and the medial thickness (Figure S9b). The proximal thickness was measured 0.5 cm from the most proximal point of the specimen, perpendicular to the axis of percussion. The distal thickness was measured 0.5 cm from the most distal point of the specimen, also perpendicular to the axis of percussion. The medial thickness is the same measurement as the thickness measurement presented in Figure S8.

Once all three thicknesses were measured they were ordered from least to greatest. For instance, a specimen with a proximal thickness of 1.40 cm, a medial thickness of 1.60 cm, and a distal thickness of 1.55 was categorized as “proximal-distal-medial” (“pdm”). Each specimen was assigned a number representative of each possible categorization:

Distal-medial-proximal (dmp) = 1 (distal is least thick, then medial, then proximal)

Distal-proximal-medial (dpm) = 2

Medial-distal-proximal (mdp) = 3

Medial-proximal-distal (mpd) = 4

Proximal-distal-medial (pdm) = 5

Proximal-medial-distal (pmd) = 6

In the event of a tie between two section thicknesses, the following procedure was implemented:

(1) In the event of a tie between the proximal and medial section thicknesses, the proximal was categorized as being less thick.

(2) In the event of a tie between the proximal and distal section thicknesses, the proximal section was categorized as being less thick.

(3) In the event of a tie between the medial and distal section thicknesses, the medial section was categorized as being less thick.

S3.1.4. Tool class examples

Once a specimen was assigned a number from each of the three tool modes, the numbers were put together to form a tool morphological class. Thus, a pencil-like specimen with a width category of dmp and a thickness category of dmp would be “111” (Figure S10a). A coin-like specimen with a width category of pmd and a thickness category of pdm would be “265” (Figure S10b). In total, there were 108 possible tool morphological classes (three mode 1 possibilities multiplied by six mode 2 possibilities multiplied by six mode 3 possibilities).

S3.2.1. The construction of edge morphological classes

Edge morphological classes were created per tool “section,” that is the distal tool section, proximal, left lateral, and right lateral (Figure S11). Thus, an unbroken unifacially flaked stone tool contributed four edge specimens to the total pool. Broken tools may have contributed three, two, or one edge specimens, depending on the amount of fragmentation.

Four modes were used for the creation of edge classes: edge angle, edge shape, notch presence, and spur presence. These dimensions were chosen because together they capture most of an edge’s morphology, and are commonly tallied traits on Paleoindian unifacial stone tools. What follows is the procedure for their measurement and implementation in edge class construction.

S3.2.2. Mode 1: edge angle

Edge angle was measured with a pair of Baseline Stainless long-armed goniometers (Figure S12c). For each edge, edge angle was measured at three spots (Figure S12a, b). These three measurements were then averaged for the calculation of an gross edge angle value. Gross edge angle values between 0 and 30 were scored as “1”; 31 and 60 were scored as “2”; 61 and 90 were scored as “3”; over 90 were scored as “4”.

S3.2.3. Mode 2: edge shape

Edge shape was calculated using Collin’s (1999) Index of Curvature (IC). This Index divides the value “b” which is “the maximum perpendicular distance between that plane and the interior surface of the blade” by the value “a” of the “straight-line distance between the distal and proximal points of contact of the interior blade surface and a flat plane” (Collins 1999: 86, see Figure S13).

S3.2.4. Mode 3: notch presence

A notch was defined as any section of the edge less than 12 mm in length that possessed a concavity of more than 1.5 mm (Figure S14). A box of these dimensions was drawn and potential notches were fit into it in order to determine whether they met the metric requirements. A score of “1” indicated the presence of a single notch on an edge; “2” indicated two or more notches present on the edge; “3” indicated the absence of edge notches.

The values of 12 mm and 1.5 mm were arbitrarily decided upon as the standard of notch dimensions after examination of the Paleo Crossing unifacially flaked stone tools. This was because notches inferred to be intended as such (via retouch, i.e. secondary modification) generally fell within these parameters. Of course, there is no way of confirming prehistoric intention and surely a small number of “real” and “false” notches were wrongly identified. But the purposes of quantification and measurement standardization were deemed to be more important than attempting to identify something impossible to confirm.

S3.2.5. Mode 4: spur presence

A spur was defined as any projection no wider than 3 mm, but is at least 1 mm long (Figure S15). A score of “1” indicated the presence of a single spur; “2” indicated two or more spurs present on an edge; “3” indicated the absence of spurs.

The values of 3 mm and 1mm were arbitrarily decided upon as the standard of spur dimensions after examination of the Paleo Crossing unifacially flakes stone tools for the same reasons as give in the last section.

S3.2.6. Edge class examples

Once a specimen was assigned a number from each of the four edge modes, the numbers were put together to form an edge morphological class. For example, an edge specimen with an edge angle of 23, a convex shape, with one notch and one spur would be “1111” (Figure S16a). An edge specimen with an edge angle of 62, a straight shape, with two notches and no spurs would be “2323” (Figure S16b). In total, there were 108 possible edge morphological classes (four mode 1 possibilities multiplied by three mode 2 possibilities multiplied by three mode 3 possibilities multiplied by three mode 4 possibilities).

References:

Collins M (1999) Clovis Blade Technology. Austin: University of Texas Press.

Dunnell R (1971) Systematics in Prehistory. New York: The Free Press.

Supplementary Figure Captions

Figure S1. Unifacial stone tools from the site of Paleo Crossing, Ohio. (Photograph by Elizabeth Russell)

Figure S2. Unifacial stone tools from the site of Paleo Crossing , Ohio. (Photograph by Elizabeth Russell)

Figure S3. Unifacial stone tools from the site of Paleo Crossing , Ohio. (Photograph by Elizabeth Russell)

Figure S4. An example of how a unifacial stone tool might be held by the hand for hide-scraper. The tool’s flake scars have been outlined in black.

Figure S5. An example of how a unifacial stone tool could be hafted into a wooden handle. Dorsal view (left), right lateral edge (right). Accessed from the Lithic Casting Lab, 26 October 2011 (<http://lithiccastinglab.com/gallery-pages/pointendscrapersthulelarge.htm>)

Figure S6: The three requirements for the identification of a unifacial stone tool are that there is retouch on the specimen’s dorsal face (a); that the intersection of the ventral and dorsal faces (b2) is no more than one third (b1 to b3) of the specimen thickness (b1 to b4); and that if there is retouch on the ventral face, it must not cover more than 50% of that face (c).

Figure S7. This is the triangular coordinate graph of Collins (1999), with three examples falling in three different segments of the graph. The wire-outlines above or below each unifacial stone tool image are the cross-sections of the specimen’s thickness.

Figure S8. A unifacial stone tool showing how length, width, and thickness was measured.

Figure S9. A unifacial stone tool showing how the “width category” (a) and the “thickness category” (b) were measured.

Figure S10. Two schematic examples of the two unifacial stone tool morphological classes described in the text: a pencil-like specimen with a width category of dmp and a thickness category of dmp (a); A coin-like specimen with a width category of pmd and a thickness category of pdm (b).

Figure S11. Examples of unifacial tool sections: the distal edge section, proximal edge section, left edge section, and right edge section.

Figure S12. Two examples of where edge angles were measured on each tool section mode (a, b). Edge angle measurements were taken with long-armed goniometers (c).

Figure S13. Two examples demonstrating how edge shape was determined. In each case the short distance “b” was divided by the long distance “a”.

Figure S14. Two examples of how the presence of notches was determined. A box of 12 mm by 1.5 mm was constructed (a). A notch was any section of the edge less than 12 mm in length that possessed a concavity of more than 1.5 mm (b) (compare with c).

Figure S15.Two examples of how the presence of spurs was determined. A box of 3 mm by 1 mm was constructed (a). A spur was any projection no wider than 3 mm, but at least 1 mm long (b) (compare with c).

Figure S16. Two examples of edge morphological classes: an edge specimen with an edge angle of 23, a convex shape, with one notch and one spur (a); an edge specimen with an edge angle of 62, a straight shape, with two notches and no spurs (b).
