# Supplementary material for: Estimating the Richness of a Population When the Maximum Number of Classes Is Fixed: A Nonparametric Solution to an Archaeological Problem
Source: PLoS One. 2012 May 29;7(5):e34179. doi: 10.1371/journal.pone.0034179 (PMC3362599; doi:10.1371/journal.pone.0034179)
Supplement: Appendix S1 — An illustrative example for calculating doubly-bound confidence intervals. (DOC) [file pone.0034179.s018.doc]

Appendix: Calculation Details

We use an example to illustrate how to calculate a confidence interval using our new method. The following calculation is obtained by an Excel calculation sheet, which is available in the supplementary materials or upon request from AC. Consider the edge class data for the Udora site (Table 2). The reference sample size is n = 634, and the observed richness S_obs_ = 37. Fifteen classes were each represented once and three classes appeared twice in the reference sample. Here we have f_1_ = 15, f_2_ = 3, U =108. The step-by-step calculation details follow here:

(1) From Eq. (1), we first obtain the Chao1 estimate: = 37 +225/6 = 74.50.


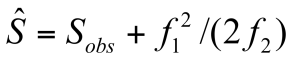


(2) Based on Eq. (2), a variance estimator of the Chao1 estimator can be computed: = 881.25.


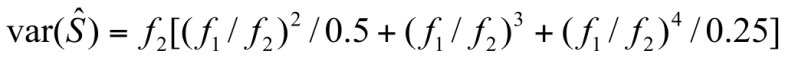


(3) Then we have = 0.4865 and σ = 0.6975 from Eq. (3). Thus, from Eq. (4), we obtain a 95% confidence interval of [46.56, 184.15], for which the lower limit is greater than the observed richness. But the upper limit exceeds the maximum possible value of 108, which we adjust in the next step.


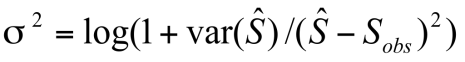


(4) We calculate = 3.6243, and = 0.82. Setting α = 0.05, we have zpα/2 = − 2.0436 and zp(1−α/2) = 0.8397. Therefore, the 95% confidence interval for S based on Equation (5) is


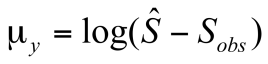

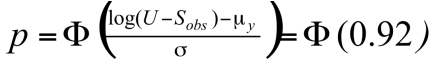

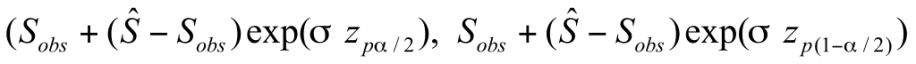


= (37 + 37.5 exp(−0.69752.0436), 37 + 37.5 exp(0.6975×0.8397))

= (46.02, 104.36).

Now the new lower bound is still greater than S_obs_ and the new upper bound is less than U = 108. Similar calculation steps can be used to obtain confidence intervals for the expected rarefied class richness (Eq. 8) and the expected extrapolated class richness (Eq. 7).
